# Supplementary material for: The Prognostic Biomarkers of Plasma Trimethylamine N-Oxide and Short-Chain Fatty Acids for Recanalization Therapy in Acute Ischemic Stroke
Source: Int J Mol Sci. 2023 Jun 28;24(13):10796. doi: 10.3390/ijms241310796 (PMC10342175; doi:10.3390/ijms241310796)
Supplement: Supplementary file 1 [file ijms-24-10796-s001.zip › ijms-2441853-supplementary.pdf]

Table S1. Details of recanalization therapy of study participants stratified by functional outcomes.

Characteristics	Total	Mild to moderate disability (n =23)	Severe disability (n =33)	p value
Recanalization therapy, n (%)				0.006
IVT only	30 (53.6%)	18 (78.3%)	12 (36.4%)	
EVT only	15 (26.8%)	4 (17.4%)	11 (33.3%)	
Both IVT and EVT	11 (19.6%)	1 (4.3%)	10 (30.3%)	
Onset to IVT time, minutes, mean (±SD) (n = 41)	133.8±50.4	137.5±58.1 (N=19)	130.6±43.9 (N=22)	0.667
Onset to puncture time, minutes, mean (±SD) (n = 26)	331.2±120.5	278.6±101.9 (N=5)	343.8±123.4 (N=21)	0.286
Onset to reperfusion time, minutes, mean (±SD) (n =2 6)	375.2±124.7	305.0±115.8 (N=5)	391.9±123.5 (N=21)	0.166
Onset to IVT time, median minutes, IQR (n = 41)	119 (95-165)	110 (95-199)	121 (95.8-153.3)	0.630
Onset to puncture time, median minutes, IQR (n = 26)	305 (254.5-377)	255 (185-384)	305 (275.5-379)	1.000
Onset to reperfusion time, median minutes, IQR (n = 26)	370.5 (283.8-451.8)	280 (202-420.5)	388 (310-453.5)	0.322
mTICI ≥ 2b, n (%) (n = 26)	23 (88.5%)	5 (100.0%)	18 (85.7%)	0.369
Occluded vessel, n (%)				0.723
Anterior circulation	45 (80.4%)	19 (82.6%)	26 (78.8%)	
Posterior circulation	11 (19.6%)	4 (17.4%)	7 (21.2%)	

Abbreviations: EVT, endovascular thrombectomy; IQR, interquartile range; IVT, intravenous thrombolysis; mTICI, modified treatment in cerebral infarction; SD, standard deviation.

Table S2. Differences in the plasma levels of TMAO and SCFAs between sex and vascular risk factors.

		N	TMAO (μM)		Formate (μM)		Acetate (μM)		Isobutyrate (μM)		Isovalerate (μM)	
			mean (±SD)	p value	mean (±SD)	p value	mean (±SD)	p value	mean (±SD)	p value	mean (±SD)	p value
Sex	Female	24	1.1±1.2	1.000	75.5±16.9	0.898	143.9±64.7	0.399	18.1±5.7	0.479	1.5±0.6	0.090
	Male	32	1.1±1.1		74.9±17.3		130.3±54.4		16.9±6.4		1.8±0.8	
HTN	No	10	1.2±1.5	0.611	85.5±23.8	0.033	155.6±49.2	0.248	15.9±6.3	0.416	1.9±1.1	0.448
	Yes	46	1.0±1.0		73.0±14.5		131.6±60.3		17.7±6.1		1.6±0.6	
Diabetes mellitus	No	33	1.0±1.0	0.440	77.2±19.6	0.282	134.4±58.7	0.816	17.8±6.1	0.544	1.7±0.8	0.728
	Yes	23	1.2±1.3		72.2±12.1		138.2±60.1		16.8±6.2		1.6±0.7	
Hyperlipidemia	No	14	1.0±1.1	0.978	78.6±15.6	0.386	148.9±66.5	0.370	20.9±7.1	0.012	1.6±0.9	0.647
	Yes	42	1.1±1.1		74.0±17.5		132.0±56.4		16.2±5.3		1.7±0.7	
Atrial fibrillation/flutter	No	29	1.0±1.1	0.734	76.5±20.0	0.556	134.8±56.5	0.872	18.2±6.7	0.312	1.9±0.8	0.014
	Yes	27	1.1±1.1		73.8±13.3		137.4±62.3		16.5±5.4		1.4±0.5	

Abbreviations: SCFA, short-chain fatty acids; SD, standard deviation; TMAO, trimethylamine N-oxide.

Table S3. Pearson correlation among patient demographics, laboratory results, and plasma TMAO and SCFA levels.

	TMAO (μM)		Formate (μM)		Acetate (μM)		Isobutyrate (μM)		Isovalerate (μM)	
	Pearson's r	p value	Pearson's r	p value	Pearson's r	p value	Pearson's r	p value	Pearson's r	p value
Age	0.166	0.220	0.148	0.278	0.137	0.317	0.019	0.888	-0.028	0.835
BMI	-0.166	0.231	-0.107	0.442	-0.319	0.020	0.033	0.812	-0.130	0.349
TC	-0.130	0.340	-0.012	0.932	-0.289	0.032	-0.140	0.302	0.195	0.151
HDL-C	0.118	0.386	0.111	0.415	0.051	0.710	0.007	0.958	0.057	0.677
LDL-C	-0.126	0.356	0.042	0.758	-0.275	0.042	-0.144	0.289	0.209	0.122
TG	-0.134	0.325	-0.230	0.088	-0.216	0.113	0.085	0.535	-0.063	0.646
HbA1c	0.400	0.002	-0.056	0.684	-0.030	0.830	-0.177	0.191	-0.022	0.875

Abbreviations: BMI, body mass index; HbA1c, glycated hemoglobin; HDL-C, high-density lipoprotein cholesterol; LDL-C, low-density lipoprotein cholesterol; SCFA, short-chain fatty acids; TC, total cholesterol; TG, triglyceride; TMAO, trimethylamine N-oxide.

Methods for measurement of TMAO

Liquid chromatography–mass spectrometry (LC–MS)
For all detection and quantification of analytes, Waters ACQUITY UPLC system (Waters Corporation, Milford, MA) coupled with a tandem MS (Finnigan TSQ Quantum Ultra triple-quadrupole MS, Thermo Electron, San Jose, CA) in combination with the Xcalibur software (ThermoFinnigan, Bellefonte, PA) was used. The LC–MS–MS system was equipped with an electrospray ion source (ESI) and was running in positive mode. The injection volume was 10 µL on a ACQUITY UPLC BEH C18 Column (130Å, 1.7 µm, 2.1 mm X 50 mm, Waters Corporation, Milford, MA) equipped with a filter (Waters Acquity UPLC™ BEH C_{18} column,1.7µm,2.1mm×5mm) in front of the column.

The flow rate was 250 µL/min, and the column temperature was 40°C. Solvents were A: 0.1% formic acid in water and B: 0.1% formic acid in acetonitrile.
Solvent programming was 0.0–0.5min, 1% B; 4 min, 50% B;4.1–5.0 min, 1% B.

MS–MS interphase settings used were as follows: spray voltage, 3000 V; sheath gas (N2) pressure, 28 psi; auxiliary gas (N2) pressure, 10psi; capillary temperature, 350°C; collision gas (Ar) pressure,1.0 mTorr. Precursor/product ions in positive mode $[M+H]^+$, retention time of the analytes separated on the LC column, and the individual collision energies, and the tube lens for the formation of product ions are listed in Table S4. The parameters of calibration curve for the measurement of TMAO are listed in Table S5.

Table S4. TMAO, positive and labeled precursor and product ion transitions monitored, collision energies, retention times, and tube lens.

	Precursor/ product ions (*m*/*z*)	Isotope-labeled precursor/ product ions (*m*/*z*)	Retention time (min)	Collision energy (V)	Tube lens (V)
TMAO	76/58	85/69 d9-TMAO	0.96	20	69

Table S5. The parameters of calibration curve for the measurement of TMAO.

Calibration curve	Unit: ppb
Range	1ppb~250ppb
Coefficient of determination	R^2=0.9990
Limit of quantitation	0.5ppb
Retention time (min)	0.96

Preparation of serum and standards:

1. Serum was thawed and 50 μL was transferred to a 2-mL Eppendorf tube.
2. Internal standard solution containing the labelled internal standards (10 Ml)(d9-TMAO (20ppb) in LC-MS grade acetonitrile (150 μL) was added to the serum.
3. The tube was vortexed for 30 s and centrifuged at 4000 rpm and 4 °C for 10 min.
4. For LC-MS analysis using the ACE C18-PFP column, an aliquot (100 μL) of supernatant was transferred to a 2-mL Eppendorf tube and dried down using a Speedivac, then reconstituted with 100 μL LC-MS grade water containing 0.1% FA.
5. The final volume after pretreatment of 50ul serum is 100ul. Therefore, the volume is diluted by 2 times, so the actual concentration should be increased by 2 times

Methods for measurement of SCFAs

Liquid chromatography–mass spectrometry (LC–MS)
For all detection and quantification of analytes, Waters ACQUITY UPLC system (Waters Corporation, Milford, MA) coupled with a tandem MS (Finnigan TSQ Quantum Ultra triple-quadrupole MS, Thermo Electron, San Jose, CA) in combination with the Xcalibur software (ThermoFinnigan, Bellefonte, PA) was used. The LC–MS–MS system was equipped with an electrospray ion source (ESI) and was running in negative mode. The injection volume was 10 µL on a ACQUITY UPLC BEH C18 Column (130Å, 1.7 µm, 2.1 mm X 100 mm, Waters Corporation, Milford, MA)equipped with a filter (Waters Acquity UPLC™ BEH C_{18} column,1.7µm,2.1mm×5mm) in front of the column.

The flow rate was 300 µL/min, and the column temperature was 40°C. Solvents were A: 0.1% formic acid in water and B: 0.1% formic acid in acetonitrile.
Solvent programming was 0.0–3.0min, 10% B; 17.0 min, 55% B;17.1–18.1 min, 100% B; 18.5–20.0 min, 10% B.

MS–MS interphase settings used were as follows: spray voltage, 3000 V; sheath gas (N2) pressure, 45 psi; auxiliary gas (N2) pressure, 10psi; capillary temperature, 350°C; collision gas (Ar) pressure,1.0 mTorr. Precursor/product ions in negative mode $[M-H]^-$, retention time of the analytes separated on the LC column, and the individual collision energies, and the individual tube lens for the formation of product ions are listed in Table S6. The parameters of calibration curve for the measurement of SCFAs are listed in Table S7.

Table S6. SCFAs, native and labeled precursor and product ion transitions monitored, collision energies, retention times, and tube lens.

	Precursor/ product ions (*m*/*z*)	Isotope-labeled precursor/ product ions (*m*/*z*)	Retention time (min)	Collision energy (V)	Tube lens (V)
Formate	180/137	186/143	4.60	20	29
Acetate	194/137	200/143	5.10	23	39
Propionate	208/137	214/143	7.10	29	41
Isobutyrate	222/137	228/143	8.79	21	40
Butyrate	222/137	228/143	9.07	21	40
2-methylbutyrate	236/137	242/143	10.50	21	40
Isovalerate	236/137	242/143	10.76	23	50
Valerate	236/137	242/143	11.13	23	50
3-methylavalerate	250/137	256/143	12.53	33	50

Table S7. The parameters of calibration curve for the measurement of SCFAs.

Calibration curve	Formate	Acetate	Propionate	Isobutyrate	Butyrate	2-methylbutyrate	Isovalerate	Valerate	3-methylavalerate
Range	10uM~250uM	25uM~2500uM	0.5uM~50uM	0.1uM~5uM	0.1uM~50uM	0.1uM~5uM	0.1uM~5uM	0.1uM~5uM	0.1uM~5uM
Coefficient of determination	R^2=0.9978	R^2=0.9992	R^2=0.9988	R^2=0.9998	R^2=0.9997	R^2=0.9995	R^2=0.9999	R^2=0.9997	R^2=0.9997
Limit of quantitation	0.5uM	1uM	0.5uM	0.1uM	0.1uM	0.1uM	0.1uM	0.05uM	0.1uM
Retention time (min)	4.57	5.21	7.08	8.78	9.07	10.49	10.78	11.14	12.54

For derivatization, 50μL human serums were mixed with 20μL of 200 mM 3NPH in 50% aqueous acetonitrile and 20μL of 120 mM EDC-6% pyridine solution in the same solvent. The mixture was reacted at 40℃ for 30 min. After reaction, this solution was diluted to 300μL with 10% aqueous acetonitrile. A 75μL aliquot was mixed with 25μL of the IS mix, and a 10 μL aliquot was injected for LC-MS/MS

3-Nitrophenylhydrazine hydrochloride: (3NPH)_HCl
N-(3-Dimethylaminopropyl)-*N′*-ethylcarbodiimide hydrochloride: (EDC)_HCl

Isotope-labeled IS mix
50μL of a mixed standard solution containing 4 mM of acetic acid, 2 mM of propionic acid, and 1 mM of each of the other seven SCFAs were added to a 3-mL borosilicate test tube that contained 1 mg of 13C6-3NPH_HCl. 20μL of 120 mM EDC solution in 50% aqueous acetonitrile and 20μL of 6% pyridine in the same solvent were then added to the mixture. The mixture was reacted at 40 ℃ for 30 min. After reaction, the mixture was transferred to a volumetric flask with 10% aqueous acetonitrile and diluted with the same solvent to 100 mL. This solution was used as the IS mix. This solution was stable for at least three months when stored at -20 ℃.
